# Association between biofilm formation phenotype and clonal lineage in *Staphylococcus aureus* strains from bone and joint infections

**DOI:** 10.1371/journal.pone.0200064

**Published:** 2018-08-30

**Authors:** Jason Tasse, Sophie Trouillet-Assant, Jérôme Josse, Patricia Martins-Simões, Florent Valour, Carole Langlois-Jacques, Stéphanie Badel-Berchoux, Christian Provot, Thierry Bernardi, Tristan Ferry, Frédéric Laurent

**Affiliations:** 1 Centre International de Recherche en Infectiologie, INSERM U1111, Pathogénie des Staphylocoques, Lyon, France; 2 Université Claude Bernard Lyon 1, Lyon, France; 3 Institut des Agents Infectieux, Hospices Civils de Lyon, Lyon, France; 4 BioFilm Control, Saint-Beauzire, France; 5 Service des Maladies Infectieuses et Tropicales, Hospices Civils de Lyon, Lyon, France; 6 Service de Biostatistique et Bioinformatique, Hospices Civils de Lyon, Lyon, France; 7 Laboratoire de Biométrie et Biologie Evolutive, Equipe Biostatistique-Santé, CNRS, UMR, Villeurbanne, France; Institut Pasteur, FRANCE

## Abstract

Biofilm formation is a critical virulence factor responsible for treatment failure and chronicity in orthopaedic device-related infections (ODIs) caused by *Staphylococcus aureus*. Clonal lineages differ in terms of their biofilm forming capacities. The aim of this study was to investigate the correlation between the clonal complex (CC) affiliation and biofilm phenotype of 30 clinical *S*. *aureus* isolates responsible of ODI based on i) early biofilm formation using BioFilm Ring Test® and mature biofilm formation using crystal violet assays, ii) biofilm composition using DNase and proteinase K activity, and iii) prevention of biofilm formation by cloxacillin, teicoplanin and vancomycin using Antibiofilmogram® (biofilm minimal inhibitory concentration–bMIC). In terms of early biofilm formation, the CC30 strains were significantly slower than the CC5, CC15 and CC45 strains. CC45 strains produced significantly more mature biofilm than other group of strains did. The formation of biofilms was highly dependent on the presence of extracellular DNA in the CC5, CC15 and CC30 strains whereas it was mostly dependent on the presence of proteins in CC45. Finally, the CC30 group highlighted higher proportion of susceptible (bMIC < breakpoints of EUCAST guidelines) for cloxacillin, teicoplanin and vancomycin compared to the other CCs. These results demonstrate that the biofilm phenotype of clinical *S*. *aureus* isolates from ODIs is strongly related to their respective CC affiliation.

## Introduction

Orthopaedic device-related infections (ODIs) are considered as difficult to treat complications involving prosthetic devices [1]. Surgical intervention by debridement-lavage or prosthesis replacement is typically required, in association with prolonged antimicrobial therapy [2,3]. *Staphylococcus aureus* (*S*. *aureus*) is the cause of approximately one third of all ODIs [4,5]. Even with optimal management, treatment failure or infection relapses occurred in 10 to 20% of patients [6]. These complications often result from the formation of bacterial biofilms [7–9].

A biofilm is a structured community of bacteria attached to a biotic or abiotic surface and encased in a matrix of polysaccharides, proteins, extracellular DNA (eDNA) and host components [10,11]. Within the biofilm, bacteria are protected from the host immune system and exhibit phenotypic antibiotic tolerance [12]. Classically, the minimal inhibitory concentration (MIC) is determined for clinical isolates involved in infections and is used to choose and optimize the antimicrobial chemotherapy. However, this approach only probes the antimicrobial activity against planktonic bacteria and does not take into account their sessile forms. Hence, classical MICs are not predictive of the efficacy of antibiotics against biofilms synthesis or eradication. In a previous study, we reported that the biofilm MIC (bMIC) corresponding to the antibiotic concentration that prevents biofilm establishment, can be assessed using new Antibiofilmogram® tests [13]. Measuring bMIC is a promising *in vitro* parameter to predict the ability of antibiotics to prevent *in vivo* biofilm formation in clinical infection. We found that cloxacillin, teicoplanin and vancomycin have strain-dependent bMIC distribution patterns [11]. However, the ability of certain strains to form biofilms or not in the presence of a specific concentration of antimicrobials remained not explained at the time. We hypothesized that this might be due to specificities of the biofilms' phenotypes (i.e. qualitative, quantitative and/or dynamic features) related to the clonal lineage of the strains. The aim of the present study was to investigate the possible link between clonal lineage and biofilm phenotype using a collection of 30 clinical *S*. *aureus* strains responsible for ODIs.

## Material and methods

### *S*. *aureus* strains

Thirty strains of methicillin-susceptible *S*. *aureus* were selected from a collection of isolates responsible for a well-documented first episode of monomicrobial ODI at the Croix Rousse University Hospital in Lyon, France, from 2001 to 2010. Patients with clinical evidence of infection and at least 2 deep bacteriological *S*. *aureus*-positive samples were considered for inclusion. The collection of clinical data and clinical strains was approved by the French South-East ethics committee (reference number 2013–018). All the strains were characterized using an Alere StaphyType DNA microarray (StaphyType; Clondiag, Germany) [14]. The isolates were assigned to a clonal complex (CC) by comparison with a database of reference strains previously subjected to multi-locus sequence typing (MLST). Finally, all strains were susceptible to cloxacillin and glycopeptides based on the E-test® method (bioMérieux, France).

Antibiofilmogram® was previously performed to determine the capacity of 11 antibiotics to prevent biofilm formation [13]. Briefly, bacteria were cultured in microplate in presence of BHI containing antibiotic and micromagnetic beads. After 4 hours, microplate was placed on a magnetic block where, in the absence of biofilm, the free magnetic beads were attracted to the center of the well and form a visible spot. Conversely, in the presence of biofilm, the beads were trapped and only a weak spot (or none) appears. Using this method and the MIC breakpoint defined in the 2015 EUCAST guidelines, the strains were classified as bMIC resistant (bMIC-R) if the bMIC was higher than the breakpoint and as bMIC susceptible (bMIC-S) if the bMIC was lower than the breakpoint.

### Kinetic of early biofilm formation

The kinetics of early biofilm formation was assessed using BioFilm Ring Test^®^ adapted from Chavant et al. [15]. As Antibiofilmogram®, this technique involves observing the dynamic immobilization of magnetic microbeads embedded in the biofilm. Briefly, *S*. *aureus* strains stored at −20°C were subcultured on Columbia sheep blood agar (COS, bioMérieux, France) at 37°C for 24 h. A brain-heart infusion (BHI, bioMérieux, France) was then inoculated with three colonies and incubated at 37°C overnight. The bacterial suspension was standardized to an optical density at 600 nm (OD_600_) of 1.00 ± 0.05 (Ultrospec 10 Cell Density Meter, Amersham Biosciences, USA) and diluted at 1:250 in sterile BHI to obtain a final concentration of approximately 4·10^6^ UFC/mL. The bacterial suspension was supplemented with 1 vol% magnetic beads (TONER 4, Biofilm Control, France) and 200 μL were deposited in duplicate for each time point (0, 1, 2, 3, 4, 5, 6, 7 and 8 h– 37°C) in a microplate (BD Falcon 96 Flat Bottom Transparent, Corning, USA). After incubation, 100 μL of liquid contrast solution was added on the top of each well and the microplate was placed on a magnetic block for 1 min. The plates were scanned using a Biofilm Control plate reader and the intensity of the spot was analyzed and expressed as a biofilm formation index (BFI). Each observed BFI (*BFI_o_*) was normalized to a percentage of magnetic beads (Pmb) using control wells with (*BFI_ref_*) and without (*BFI_min_*) beads using the formula: $Pmb=\frac{{BFI}_{ref}-{BFI}_{o}}{{BFI}_{ref}-{BFI}_{min}}$. Each experiment was performed three times for each duplicate solution.

### Determining mature biofilm formation

The mature biofilms were evaluated using crystal violet assays [16]. The ODs_600_ of overnight bacterial suspensions were adjusted to 1 ± 0.05, before a 1:100 dilution in sterile BHI + 1% glucose (D-(+)-glucose, Biosolve, France). 100 μL samples of each suspension were transferred to a microplate (BD Falcon 96 Flat Bottom Transparent, Corning, USA) in quadruplicate and kept at 37°C for 24 h. Negative control wells were prepared with BHI alone. After incubation, microplate was washed with 200 μL of PBS 1X (Thermo Fisher Scientific, Gibco) using a pipette. This step was repeated twice. The biofilm was fixed using 100 μL of 99% methanol (VWR International, France) for 20 min. The microplates were washed again and the bacteria stained with 100 μL of 0.1% crystal violet (Merck, France) for 10 min. Excess stain was rinsed away, the dye bound to biofilm was solubilized in 100 μL of 33% acetic acid, and the OD_620_ was measured using a micro ELISA Auto Reader (Model 680, BioRad, USA). The control OD_620_ was subtracted from the OD_620_ of each tested strain. The experiment was performed three times for each sample.

### Growth rate measurement

Bacteria were grown in the presence of enzymes to confirm that the generation time was not significantly affected. Briefly, DNase (DN25, Sigma Aldrich, USA) and proteinase K (P2308, Sigma Aldrich, USA) were added (100 μg/mL and 50 μg/mL respectively) to separate bacterial suspensions standardized to 4·10^6^ CFU/mL in BHI and microplates (BD Falcon 96 Flat Bottom Transparent, Corning, USA) were filled with 200 μL of either suspension per well. Control wells were filled with BHI complemented with buffer alone. The growth rate at 37°C was monitored every 15 min for 24 h by orbital shaking and OD_600_ measurements performed using a microplate reader (Infinite® 200 PRO device Tecan, Tecan, France). The instantaneous growth rates (μ) and generation times (θ) were calculated on the basis of four consecutive OD measurements in a 1 h period according to the formula *X* = *X*_0_*e*^μ*t*^ and *θ* = ln 2/μ, where *t* is the time (h), and *X* (*X*_0_) is the biomass at time *t* (*t* = 0) as previously described [17]. For each well, the minimum calculated generation time was selected to estimate the maximal growth rate. The generation time was estimated as the mean of three experiments performed in duplicate for each sample.

### Determining the essential components for early biofilm formation

The Biofilm Ring Test^®^ method was adapted to quantify the attachment and initiation of biofilm formation in the presence of specific enzymes. Standardized bacterial suspensions containing 1 vol% magnetic beads were supplemented with DNase (100 μg/mL), proteinase K (50 μg/mL) or buffer alone and incubated at 37°C in a 96-well microplate (200 μL/well) (BD Falcon 96 Flat Bottom Transparent, Corning, USA). Negative controls consisted of 200 μL of sterile BHI containing magnetic beads and enzyme. The plate was read after 6 h of incubation, as described above. The capacity of the strains to form biofilm in the presence of enzymes was expressed using the relative difference (RD, Eq 1):

$$RD=\frac{{Pmb}_{without\;enzyme}-{Pmb}_{with\;enzyme}}{{Pmb}_{without\;enzyme}}\times 100.$$ (1)

RD = 0 indicates that the biofilms formed with and without the enzyme are equivalent, i.e. that the tested component is not involved in the initial steps of biofilm formation. Conversely, RD = 100 indicates that no biofilm forms in the presence of the enzyme, i.e. that the targeted component is essential for the initial growth of the biofilm. These measurements were performed three times in duplicate for each sample.

### Statistical analysis

Descriptive statistics were used to estimate the frequencies of the study variables, which are given as mean values and the corresponding standard errors (mean ± SEM). Differences between CC groups were assessed using the Kruskal–Wallis tests followed by the Mann-Whitney U test for biofilm experiments. Difference between bMIC distributions of the main CCs and bMIC distribution of the overall collection were assessed using the Fisher’s exact test. All analyzes were performed with Prism 5.03 software (Prism, GraphPad Software, San Diego California USA). The significance threshold used was *p* < 0.05.

## Results

### Characterization of the strains

The demographic and *S*. *aureus* ODI characteristics were detailed in Table 1. Out of the 30 clinical strains of *S*. *aureus*, 24 belonged to one of four CCs, namely CC5 (n = 6), CC15 (n = 5), CC30 (n = 7) and CC45 (n = 6) (Table 2). The remaining six were shared between CC8 (n = 2), CC22 (n = 1), CC50 (n = 1), CC88 (n = 1) and CC398 (n = 1). The lineage-dependence of the biofilm phenotype was only investigated for the four CCs with large enough sample sizes (i.e. CC5, CC15, CC30 and CC45). To assess the genetic relatedness of our collection, all strains were sequenced and SNP based phylogenetic analysis were performed for the 4 main CCs using public genomes and genomes from our collection from various origins (S1 Supplementary Information). The strains in each clonal group revealed a relative high level of heterogeneity, suggesting that they have not evolved clonally from a single clone that has disseminated. This was in accordance with the diversity of isolation date, and the community origin of infections for most of patients, or the diverse hospitals in which the nosocomial infections were accounted for the others.

**Table 1:** Clinical and demographic characteristics of the 30 included patients with *S*. *aureus* orthopaedic device-related infection.

**Demographic characteristics**	
Sex (male)	17 (56.7%)
Age (year)	52.2 (45.3–59.0)
**Orthopaedic device infection type**	
Osteosynthesis	12 (40%)
Joint prosthesis	15 (50%)
Vertebral osteosynthesis	3 (10%)
**Clinical presentation**	
Delay between implantation and infection (weeks)	134.1 (34.5–233.6)
Delay between symptoms and diagnosis (weeks)	11.8 (5.9–17.7)
Biological inflammatory syndrome	28 (93.3%)
CRP level (mg/L)	132.2 (93.9–170.4)
WBC (g/L)	10 (8.5–11.5)
**Management**	
Surgical treatment	25 (83.3%)
Antimicrobial treatment duration (weeks)	32.8 (24.8–40.7)
**Outcome**	
Treatment failure linked with the same strain	13 (43.3%)

Abbreviations: ODI, Orthopaedic Device-related Infection; CRP, C-reactive protein; WBC, white blood cell. Note: Results are presented as effective (%) or mean (95%CI) values.

**Table 2:** Virulence factor gene contents of all characterized strains grouped by clonal complex.

	CC					Total
	5	15	30	45	Other	
**No. of isolates**	**6**	**5**	**7**	**6**	**6**	**30**
*agr* type	II	II	III	I	I (n = 4),III (n = 1) IV (n = 1)	I (n = 10), II (n = 11), III (n = 8), IV (n = 1)
*cap* operon	5	8	8	8	5 (n = 4), 8 (n = 2)	5 (n = 10), 8 (n = 20)
**Biofilm formation**						
*ica* operon	+	+	+	+	+	+
*bap*	−	−	−	−	−	−
**Colonization factors**						
*bbp*	+	+	+	+ (n = 5)	+ (n = 3)	+ (n = 26)
*clf*A	+	+	+	+	+	+
*clf*B	+	+	+ (n = 6)	+	+ (n = 3)	+ (n = 26)
*can*	−	−	− (n = 4)	+	+ (n = 3)	− (n = 18)
*ebp*S	+	+	+	+	+	+
*fib*	+	+	+	+	+	+
*fnb*A	+	+	+	+	+ (n = 3)	+ (n = 27)
*fnb*B	+	+	− (n = 5)	+	+	+ (n = 25)
*sak*	+	−	+	+	− (n = 4)	+ (n = 21)
*sas*G	+	+	−	−	+ (n = 4)	+ (n = 15)
*sdr*C	+	+ (n = 4)	− (n = 6)	+	+ (n = 5)	+ (n = 22)
*sdr*D	+ (n = 5)	+ (n = 4)	+ (n = 5)	+	+ (n = 3)	+ (n = 23)
*vwb*	+	+	+	−	+	+ (n = 23)

CC: Clonal Complex; gene codings for: *agr*, accessory gene regulator; *cap*, capsular polysaccharide; *ica*, intercellular adhesion protein A; *bap*, biofilm-associated protein; *bbp*, bone sialoprotein-binding protein; *clfA*, clumping factor ClfA; *clfB*, clumping factor ClfB; *cna*, collagen-binding protein; *ebpS*, cell surface elastin-binding protein; *fib*, fibrinogen-binding protein; *fnbA*, fibronectin binding protein A; *fnbB*, fibronectin-binding protein B; *sak*, staphylokinase; *sasG*, *Staphylococcus aureus* surface protein G; *sdrC*, serine-aspartate repeat protein C; *sdrD*, serine-aspartate repeat protein D; *vwb*, secreted von Willebrand factor-binding protein precursor.

### Kinetics of biofilm formation

**Fig 1:**
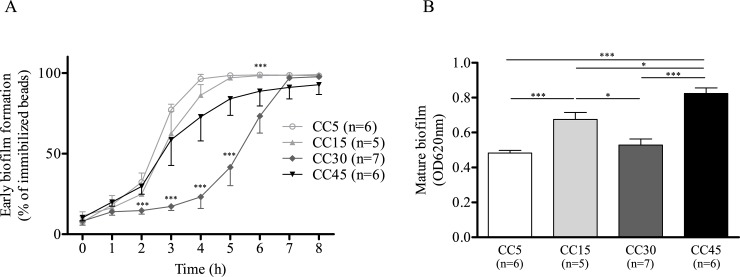
Comparison of the kinetics of biofilm formation of the main *S*. *aureus* clonal complexes (CCs). (A) Kinetics of early biofilm formation measured using BioFilm Ring Test^®^: means and corresponding standard errors of three assays of duplicate samples. (B) Amounts of biofilm formed after 24 h measured by crystal violet assays (optical density at 620 nm, OD_620_): means and corresponding standard errors of three assays of quadruplicate samples.

Although all the strains produced biofilm, the formation of early biofilm was significantly slower for the CC30 strain than it was for the CC5, CC15 or CC45 strains (Fig 1A, *p* < 0.0001 at 2, 3, 4, 5 and 6 h). After 24 h, the CC45 strain had produced significantly more mature biofilm than the CC15 strain had (Fig 1B, *p* = 0.0178), which in turn had produced significantly more than the CC5 or CC30 strains had (Fig 1B, *p* < 0.0001 and *p* = 0.0118 respectively). The difference between the amounts of biofilm produced by the latter two clones is not significant however. In summary, the CC30 and CC5 strains produced a moderate amount of mature biofilm, the former slowly and the latter rapidly. In contrast, the CC15 and CC45 strains combined rapid and extensive biofilm production.

### Biofilm composition

Since the susceptibility of a staphylococcal biofilm to enzymatic treatments depends on the composition of the biofilm, DNase and proteinase K have been proposed as means to investigate the role of eDNA and proteins, respectively, in the organization/structure of biofilm matrices [18]. The attachment and initiation of biofilm formation was investigated by measuring the RD (Eq 1) of bead immobilizations in the presence and absence of these enzymes. Note that the generation time was not significantly affected by the treatment with proteinase K or DNase I (ϴ = 25.5 ± 1.1 and 24.5 ± 1.1 min respectively, vs 27.1 ± 1.3 min without either enzyme).

**Fig 2:**
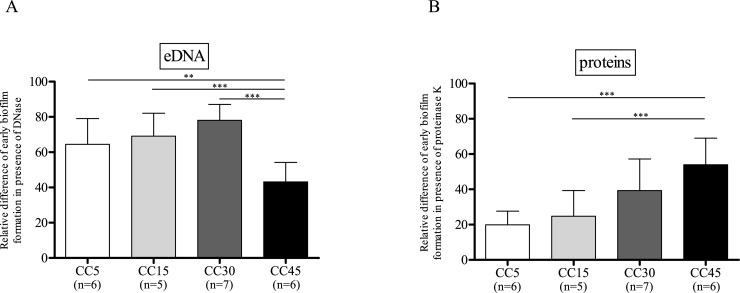
Comparison of the role of specific components on early biofilm formation for the main *S*. *aureus* clonal complexes (CCs). Results are expressed as relative differences (Eq 1) in the amounts of biofilm (measured using BioFilm Ring Tests^®^) formed after 6 h incubation in the presence of (A) DNase or (B) proteinase K versus in their absence. The values shown are means and the corresponding standard errors of three assays of duplicate samples.

The results obtained following DNAse I treatment highlight the significantly stronger implication of eDNA in biofilm growth for the CC5, CC15 and CC30 strains than for CC45 strains (Fig 2A, RD_DNAse_ = 64.5 ± 5.6, 69.1 ± 4.0 and 78.0 ± 2.8 vs 43.1 ± 3.8, p = 0.0027, p = 0.0003, p < 0.0001 respectively). Interestingly, RD_pK_ was lower than RD_DNAse_ for the CC5 and CC15 and CC30 strains (Fig 2B, RD_pk_ = 19.9 ± 3.1, 24.8 ± 4.3, 39.3 ± 6.6), but higher for the CC45 strains (RD_pk_ = 54.0 ± 5.1). Furthermore, RD_pk_ was higher for the CC45 strains than for the CC5 and CC15 strains (p < 0.0001, p = 0.0005 respectively). These results suggest that early biofilm formation depends more on eDNA than protein production for the CC5, CC15 and CC30 strains, but more on the latter for the CC45 strains.

### bMIC distributions of the main CCs

**Fig 3:**
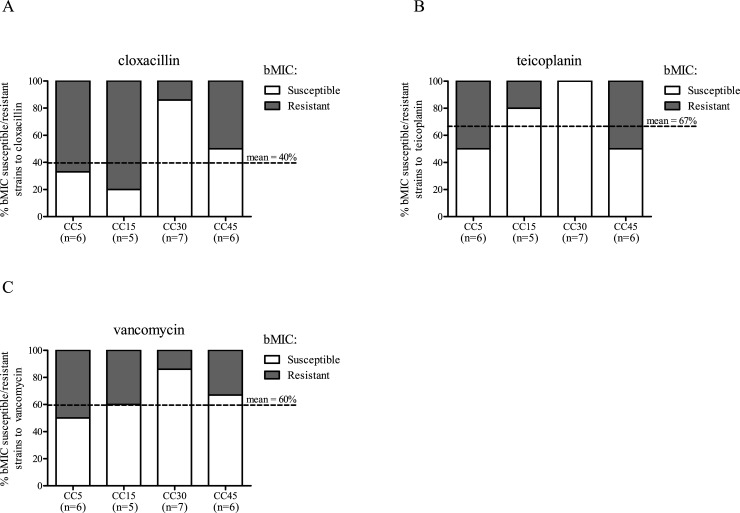
**Biofilm minimal inhibitory concentration (bMIC) susceptibility profiles of the main *S*. *aureus* clonal complexes to (A) cloxacillin, (B) teicoplanin and (C) vancomycin.** The dotted lines shown the mean percentage of susceptible strains to the corresponding antibiotic for all the CCs (n = 30).

Finally, we compared the distributions of bMIC for the different CC strains. The percentage of CC30 strains classified as bMIC-S for cloxacillin was higher than the overall mean (n = 30) (Fig 3A, 86% vs. 40%, p = 0.0422). A similar trend was also observed for this CC for teicoplanin and vancomycin (Fig 3B and 3C, 100% vs. 67% and 86% vs. 60%, p = 0.1551, p = 0.3828 respectively). The bMIC susceptibilities of the other CC strains are similar to the overall mean.

## Discussion

The presence of biofilms in ODIs is associated with treatment failure and persistence of the infection [9]. In this study, we found that the growth kinetics, composition and bMIC of biofilms of different clinical strains of *S*. *aureus* involved in ODIs depend on their CC affiliation.

Indeed, the CC30 strains have a “slow” phenotype in terms of early biofilm formation and produce moderate amounts of mature biofilm. The other CC strains (CC5, CC15 and CC45) initiate biofilm formation much faster, and produce large (CC15 and CC45) or moderate (CC5) amounts of mature biofilm. This specific behavior has already been observed by Naicker et al. who reported that CC5 and CC8 strains were greater biofilm producers [19]. The strong biofilm forming capacity of CC8 strains has also been described elsewhere [20,21]. We found only two strains belonging to CC8 in this study. This is quite surprising because CC8 is one of the most prevalent CCs in ODIs in Western Europe [22].The two CC8 strains analyzed here were only moderate biofilm producers (data not shown). Finally, our results for CC30 are in accordance with those of Cortes et al., who found that these strains produced relatively little biofilm [21].

In order to link strain phenotype with the ability to form biofilm, Bardiau et al. studied the association between biofilm formation and capsular profiles as well as *agr*-typing in *S*. *aureus* strains collected from bovine mastitis [23]. They found that the majority of high biofilm producers belong to *agr* group I [23], corresponding to CC45 in our study. However, in a further study, they reported that the majority of high biofilm producers belong to the *cap5-agrI* group [24]. The four *cap5-agrI*-type strains in our study, belonging to CC8 (n = 2), CC22 and CC398, were not particularly high biofilm producers (data not shown). In agreement with our results, Cafiso et al. reported that *agr* II (corresponding to CC5 and CC15 strains in our study) and *agr* III strains (CC30 here) respectively produce large and medium amounts of biofilm [25]. To explain the correlations between CC affiliation and biofilm phenotypes observed here, we investigate the impact of the presence/absence of biofilm-related genes and colonization factors identified using DNA microarray (Table 2) on biofilm phenotype. However, no correlation was found using this set of 170 genes and their allelic variants (data not shown), suggesting that the biofilm behavior not depend only to the presence of a specific gene, but may be the result of an association of several genes. Indeed, this set is just a part of the >3000 genes of *S*. *aureus* genomes, and further studies with extended collection are needed to correlate phenotypic and genotypic data, especially with genome-wide association studies.

Comparison between studies remains difficult because of differences in the experimental conditions used for the biofilm formation assays. Indeed, Croes et al. have shown that the glucose concentration strongly influences biofilm formation *in vitro* [20]. Specifically, they found that the higher the glucose concentration was, the more the strain qualified as a strong biofilm producer [20]. The origin of the strains also differs between studies, ranging from different human medical settings to bovine mastitis infections. Note finally that since most studies, including the present work, are monocentric, they are only representative of local epidemiological conditions.

In this study, we used BioFilm Ring Test^®^ to study the kinetics of biofilm formation. The advantage of this method is that early biofilm formation can be quantified without any washing steps [15]. It can also be used to investigate active molecules against bacterial adhesion [26–28]. Here, we used this approach to study the role of eDNA and proteins in the early stages of biofilm formation. DNase was highly effective in preventing biofilm formation in most strains except CC45, suggesting that this strain's adhesion is less dependent on eDNA. The proteinase K treatment inhibited biofilm formation less than the DNAse one did for all CCs other than CC45, suggesting that proteins play a lesser role in early biofilm formation for these strains. Interestingly, the growth of CC45 biofilms was more inhibited by DNAse than was those of the other strains, which indicates that for this CC, proteins play a pivotal role in biofilm formation. Taken together, these results suggest that the production of specific components required for early biofilm formation depends on the CC affiliation of the strain. Moreover, our study suggests that treatments targeting eDNA could prevent the formation of biofilms of clinical *S*. *aureus* strains responsible for ODIs. Note that it would be interesting to use this approach to investigate adhesion mechanisms involving polysaccharide intercellular adhesin by using enzymes that degrade polysaccharides such as dispersin B [29].

Finally, we decided to specifically focus our study to the role of proteins and eDNA on early stage of biofilm formation to correlate the role of both components with the results obtained with Antibiofilmogram®. We found that the percentage of strains classified as bMIC-S was higher for the CC30 affiliated strains than for other CCs. This specific susceptibility may be related to its low initial rate of biofilm formation and by the strong implication of eDNA in early biofilm growth. Here, we only considered the biofilm formation behavior of 30 clinical isolates. Extensive investigations of larger collections of *S*. *aureus* strains responsible for ODIs from distinct origins and comparisons with data from genome-wide association studies are required to identify the genetic regions involved in the different biofilm formation phenotypes.

## Conclusion

This study shows that the biofilm formation phenotype of *S*. *aureus* strains responsible for ODIs depends on their genetic lineage. CC30 strains were found to start forming biofilm more slowly than those of other CCs and were more susceptible to cloxacillin, teicoplanin and vancomycin in Antibiofilmogram^®^ assays. Our results also demonstrate that Biofilm Ring Tests^®^ combined with enzyme treatments can be used to explore the implication of targeted components in the initial steps of biofilm formation.

## Supporting information

S1 Supplementary Information Genetic diversity of the four main CCs. (DOC)
