# Supplementary material for: Association between biofilm formation phenotype and clonal lineage in Staphylococcus aureus strains from bone and joint infections
Source: PLoS One. 2018 Aug 30;13(8):e0200064. doi: 10.1371/journal.pone.0200064 (PMC6116976; doi:10.1371/journal.pone.0200064)
Supplement: S1 Supplementary Information — (DOC) [file pone.0200064.s001.doc]

**Material and Methods :**

Sequencing

Genomic DNA were extracted from each isolate using a QIAcube extraction kit (Qiagen). The Nextera XT DNA preparation kit (Illumina) was used to generate sequencing libraries from 1 ng of DNA. Whole-genome sequencing was finally done with an Illumina HiSeq (Illumina, San Diego, CA, USA) to generate 150-bp paired-end reads.

SNP based phylogenetic analysis

For strains belonging to CC5, CC15, CC30 and CC45 the genetic diversity between isolates was assessed using a core SNP phylogeny. Private strains from the French National Reference Center of Staphylococci and publicly available strains from the same groups were extracted from NCBI and used in the comparison. For each group the choice of publicly available genomes was performed using the following criteria: distinct countries of origin and/or distinct submitting centers; preferably complete genomes. Conserved core-genome SNPs were extracted using the Snippy v3.1 variant calling and SNP phylogeny pipeline (<https://github.com/tseemann/snippy>) with default parameters.

**
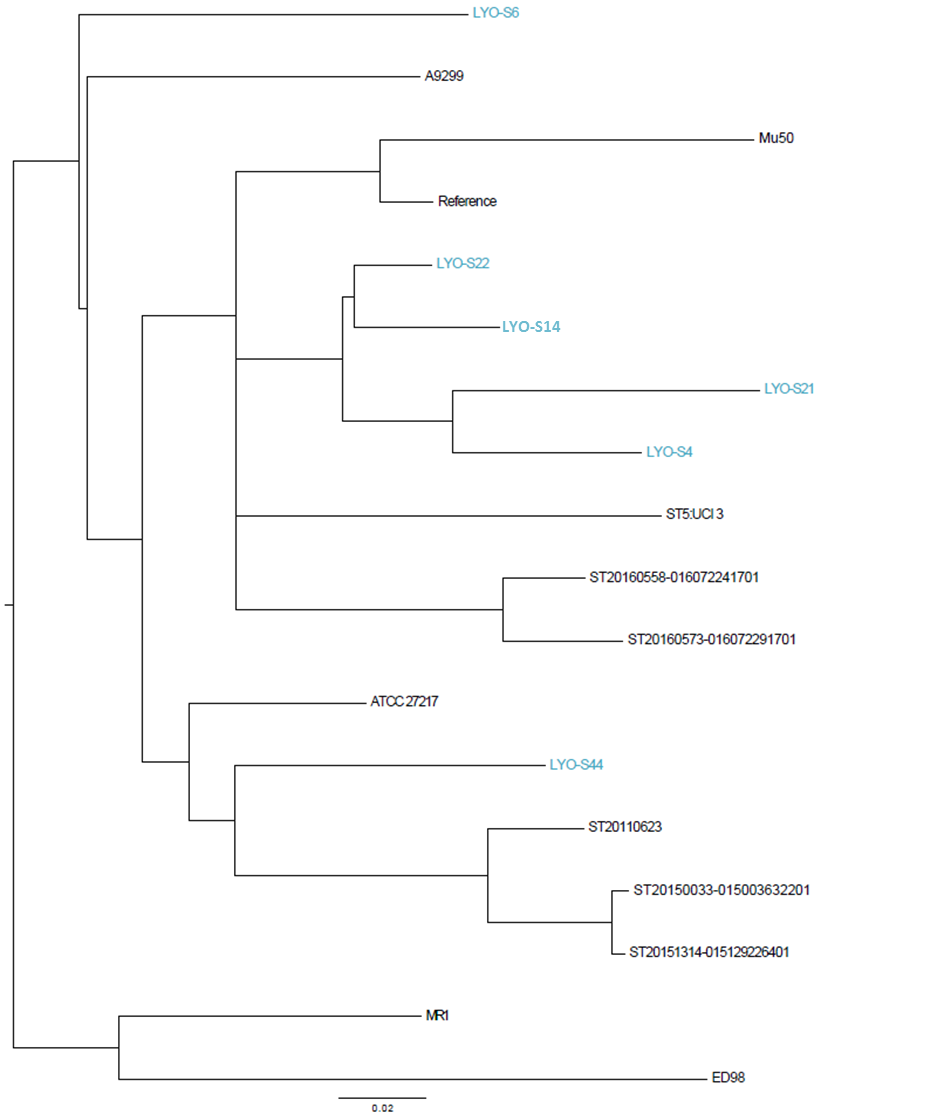
**

**Figure S1. SNP phylogeny for the CC5 group**

Isolates used in this study are colored in blue. Publicly available genomes are indicated with the strain ID as available on NCBI.


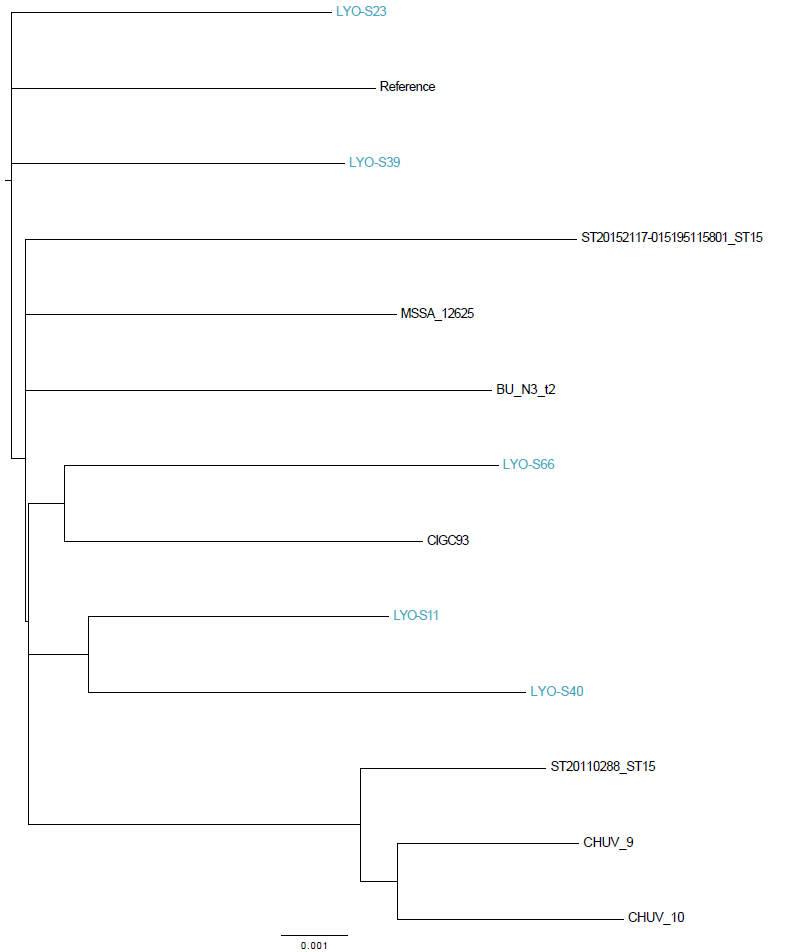


**Figure S2. SNP phylogeny for the CC15 group**

Isolates used in this study are colored in blue. Publicly available genomes are indicated with the strain ID as available on NCBI.


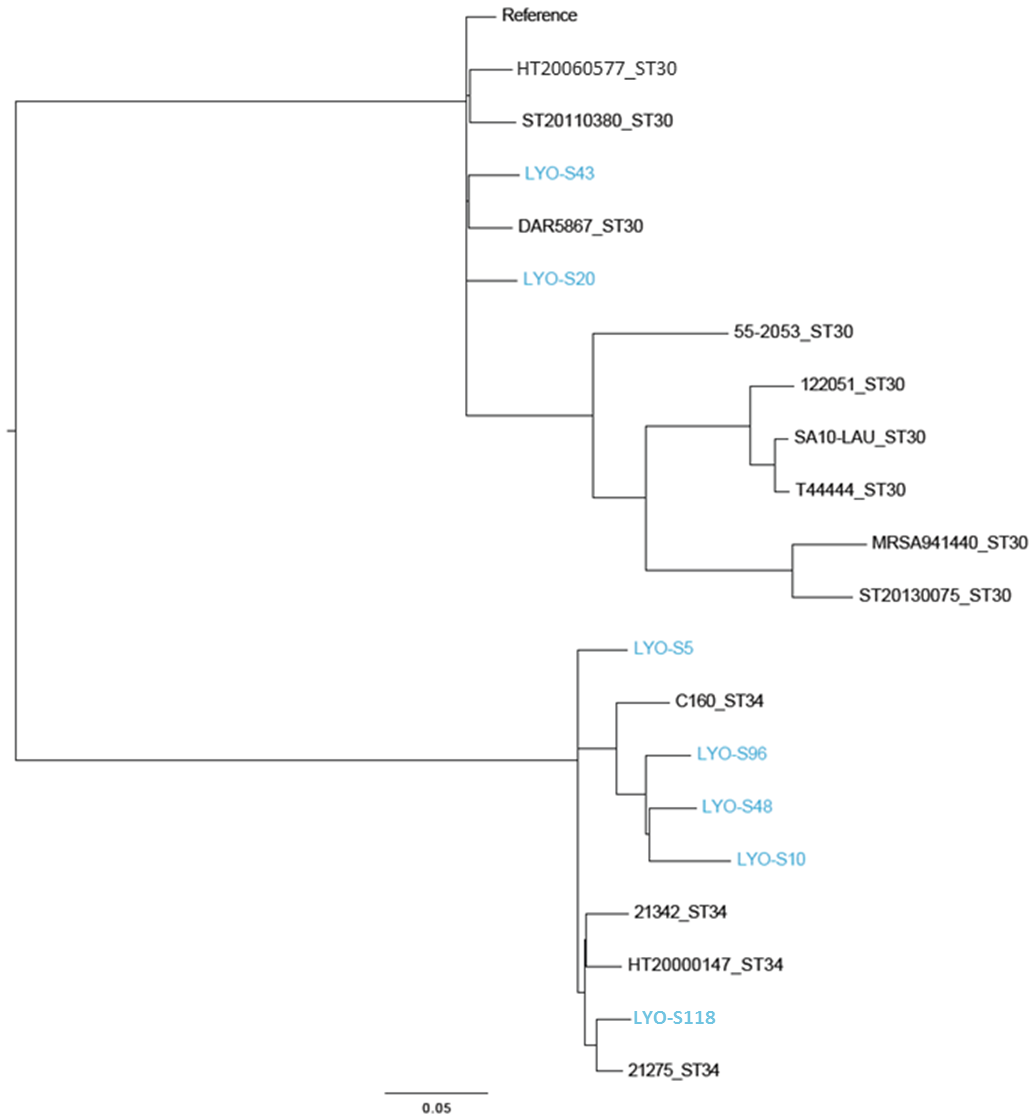


**Figure S3. SNP phylogeny for the CC30 group**

Isolates used in this study are colored in blue. Publicly available genomes are indicated with the strain ID as available on NCBI. The MLST type is indicated by “_STXX” after the name.


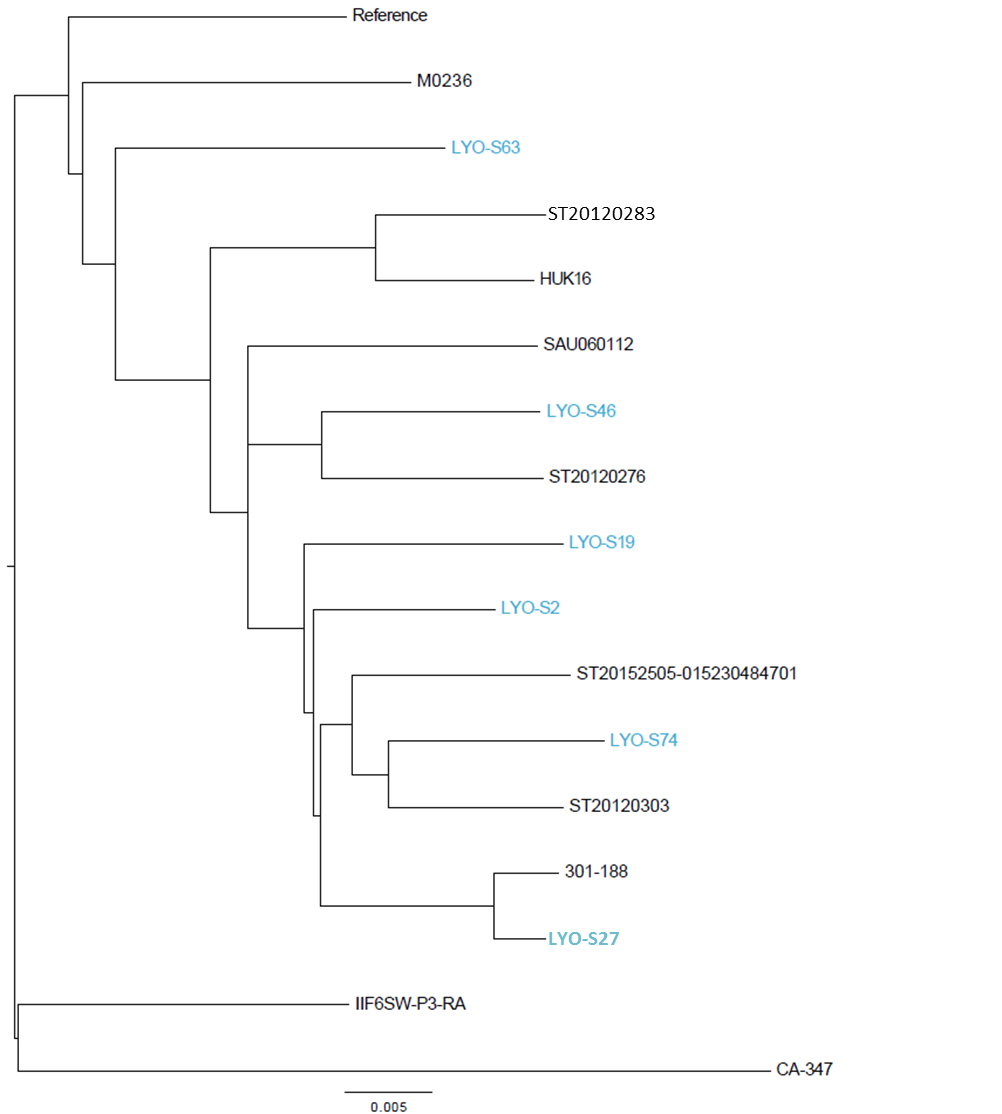


**Figure S4. SNP phylogeny for the CC45 group**

Isolates used in this study are colored in blue. Publicly available genomes are indicated with the strain ID as available on NCBI.

**Table S1. Characteristic of the private and public genomes used for the SNP based phylogenetic analysis.**

| Genome availability | ST | MRSA | Biosample | Wgs master | Infraspecific name | Assembly level | Seq rel date | Submitter | Ftp path |
| --- | --- | --- | --- | --- | --- | --- | --- | --- | --- |
| **public** | **5** | **Yes** | **SAMD00061099** |  | **N315** | **CG** | **2004/05/11** | **Juntendo Univ.** | **ftp://ftp.ncbi.nlm.nih.gov/genomes/all/GCF/000/009/645/GCF_000009645.1_ASM964v1** |
| public | 5 | No | SAMN02595322 | ACKH00000000.1 | A9299 | Contig | 2009/09/11 | Broad Institute | ftp://ftp.ncbi.nlm.nih.gov/genomes/all/GCF/000/174/555/GCF_000174555.1_ASM17455v1 |
| public | 5 | Yes | SAMN03893338 | LKYU00000000.1 | ST5:UCI 3 | Scaffold | 2017/01/05 | USDA-ARS | ftp://ftp.ncbi.nlm.nih.gov/genomes/all/GCF/001/936/695/GCF_001936695.1_UCI03_v1.0 |
| public | 5 | No | SAMN06624119 | NARB00000000.1 | ATCC 27217 | Scaffold | 2017/04/12 | Loyola University Chicago | ftp://ftp.ncbi.nlm.nih.gov/genomes/all/GCF/002/088/055/GCF_002088055.1_ASM208805v1 |
| public | 5 | Yes | SAMN02471722 | ACZQ00000000.1 | MR1 | Contig | 2009/10/26 | University of Edinburgh | ftp://ftp.ncbi.nlm.nih.gov/genomes/all/GCF/000/176/195/GCF_000176195.1_ASM17619v1 |
| public | 5 | Yes | SAMD00061098 |  | Mu50 | CG | 2004/05/14 | Juntendo Univ. | ftp://ftp.ncbi.nlm.nih.gov/genomes/all/GCF/000/009/665/GCF_000009665.1_ASM966v1 |
| public | 5 | No | SAMN02604165 |  | ED98 | CG | 2009/10/27 | University of Edinburgh | ftp://ftp.ncbi.nlm.nih.gov/genomes/all/GCF/000/024/585/GCF_000024585.1_ASM2458v1 |
| private | 5 | Yes |  |  | ST20110623 | Contig |  |  |  |
| private | 5 | Yes |  |  | ST20150033 | Contig |  |  |  |
| private | 5 | Yes |  |  | ST20151314 | Contig |  |  |  |
| private | 5 | Yes |  |  | ST20160558 | Contig |  |  |  |
| private | 5 | Yes |  |  | ST20160573 | Contig |  |  |  |
| **private** | **15** | No |  |  | **ST20111713** |  |  |  |  |
| public | 15 | No | SAMEA2384095 | FKOU00000000.1 | MSSA | Scaffold | 2016/05/19 | SC | ftp://ftp.ncbi.nlm.nih.gov/genomes/all/GCF/900/081/075/GCF_900081075.1_12625_6_63 |
| public | 15 | No | SAMN03658596 | LFNN00000000.1 | BU_N3_t2 | Contig | 2015/09/23 | Noguchi Memorial Institute for Medical Research, University of Ghana | ftp://ftp.ncbi.nlm.nih.gov/genomes/all/GCF/001/297/455/GCF_001297455.1_ASM129745v1 |
| public | 15 | No | SAMN00627583 | AHVD00000000.1 | CIGC93 | Contig | 2012/02/22 | University of Maryland | ftp://ftp.ncbi.nlm.nih.gov/genomes/all/GCF/000/249/035/GCF_000249035.1_ASM24903v2 |
| public | 15 | No | SAMEA48337918 | FUGY00000000.1 |  | Contig | 2017/02/08 | Lausanne University Hospital | ftp://ftp.ncbi.nlm.nih.gov/genomes/all/GCF/900/155/745/GCF_900155745.1_CHUV_9 |
| public | 15 | No | SAMEA48338668 | FUHJ00000000.1 |  | Contig | 2017/02/08 | Lausanne University Hospital | ftp://ftp.ncbi.nlm.nih.gov/genomes/all/GCF/900/155/795/GCF_900155795.1_CHUV_10 |
| private | 15 | No |  |  | ST20110288 | Contig |  |  |  |
| private | 15 | No |  |  | ST20152117 | Contig |  |  |  |
| **public** | **30** | **No** | **SAMN00001499** | **ACJA00000000.2** | **MN8** | **CG** | **2010/06/17** | **BCM** | **ftp://ftp.ncbi.nlm.nih.gov/genomes/all/GCF/000/160/195/GCF_000160195.1_ASM16019v1** |
| public | 30 | No | SAMN00103091 |  | 55/2053 | CG | 2013/08/21 | Broad Institute | ftp://ftp.ncbi.nlm.nih.gov/genomes/all/GCF/000/160/335/GCF_000160335.2_ASM16033v2 |
| public | 34 | No | SAMN02595353 | ACUV00000000.1 | C160 | Scaffold | 2009/12/30 | Broad Institute | ftp://ftp.ncbi.nlm.nih.gov/genomes/all/GCF/000/162/855/GCF_000162855.1_ASM16285v1 |
| public | 30 | Yes | SAMN02470734 | AHZJ00000000.1 | 122051 | Contig | 2013/05/24 | Novartis Vaccines and Diagnostics | ftp://ftp.ncbi.nlm.nih.gov/genomes/all/GCF/000/401/455/GCF_000401455.1_ASM40145v1 |
| public | 30 | Yes | SAMN02385517 | JEAR00000000.1 | T44444 | Scaffold | 2014/02/14 | Broad Institute | ftp://ftp.ncbi.nlm.nih.gov/genomes/all/GCF/000/569/635/GCF_000569635.1_Stap_aure_T44444_V1 |
| public | 30 | No | SAMN02403861 | JFRK00000000.1 | DAR5867 | Contig | 2014/03/11 | Broad Institute | ftp://ftp.ncbi.nlm.nih.gov/genomes/all/GCF/000/596/405/GCF_000596405.1_Stap_aure_DAR5867_V1 |
| public | 34 | No | SAMN00116842 | JHPV00000000.1 | 21275 | Contig | 2014/04/11 | J. Craig Venter Institute | ftp://ftp.ncbi.nlm.nih.gov/genomes/all/GCF/000/626/935/GCF_000626935.1_gsa21275v01 |
| public | 34 | No | SAMN00117467 | AHKU00000000.1 | 21432 | Contig | 2012/02/01 | J. Craig Venter Institute | ftp://ftp.ncbi.nlm.nih.gov/genomes/all/GCF/000/245/575/GCF_000245575.1_ASM24557v2 |
| public | 30 | Yes | SAMN02851473 | JHEB00000000.1 | SA10-LAU | Contig | 2014/06/12 | Lebanese American University | ftp://ftp.ncbi.nlm.nih.gov/genomes/all/GCF/000/708/405/GCF_000708405.1_Staphylococcus_aureus_SA10-LAU_WGS |
| public | 30 | No | SAMEA2298708 | FMOU00000000.1 | MRSA941440 | Scaffold | 2016/10/03 | SC | ftp://ftp.ncbi.nlm.nih.gov/genomes/all/GCF/900/097/825/GCF_900097825.1_12291_8_9 |
| private | 30 | No |  |  | HT20000147 | Contig |  |  |  |
| private | 30 | No |  |  | HT20060577 | Contig |  |  |  |
| private | 30 | No |  |  | ST20110380 | Contig |  |  |  |
| private | 30 | No |  |  | ST20130075 | Contig |  |  |  |
| **public** | **45** | **No** | **SAMN02953006** |  | **MCRF184** | **CG** | **2016/03/21** | **Research Technology Support Facility** | **ftp://ftp.ncbi.nlm.nih.gov/genomes/all/GCF/001/594/205/GCF_001594205.1_ASM159420v1** |
| public | 45 | No | SAMN02325280 | JCBG00000000.1 | M0236 | Scaffold | 2014/02/05 | Broad Institute | ftp://ftp.ncbi.nlm.nih.gov/genomes/all/GCF/000/558/485/GCF_000558485.1_Stap_aure_M0236_V1 |
| public | 45 | No | SAMN04482515 | LSMV00000000.1 | HUK16 | Contig | 2016/02/23 | Universite d'Auvergne | ftp://ftp.ncbi.nlm.nih.gov/genomes/all/GCF/001/566/905/GCF_001566905.1_ASM156690v1 |
| public | 45 | No | SAMEA2771242 | CCXN00000000.1 | SAU060112 | Contig | 2014/09/23 | Section of biotechnology | ftp://ftp.ncbi.nlm.nih.gov/genomes/all/GCF/000/824/985/GCF_000824985.1_SAU060112_PRJEB6981_wgs |
| public | 45 | Yes | SAMN02593744 | JASK00000000.1 | 301-188 | Contig | 2014/02/03 | Geneva University Hospitals, Genomic Research Laboratory. | ftp://ftp.ncbi.nlm.nih.gov/genomes/all/GCF/000/534/835/GCF_000534835.1_Saureus301-188 |
| public | 45 | No | SAMN04902357 | MIZS00000000.1 | IIF6SW-  P3-RA | Contig | 2016/09/24 | Jet Propulsion Laboratory, California Institute of Technology | ftp://ftp.ncbi.nlm.nih.gov/genomes/all/GCF/001/743/275/GCF_001743275.1_ASM174327v1 |
| public | 45 | Yes | SAMN02603909 |  | CA-347 | CG | 2013/06/20 | TGen North | ftp://ftp.ncbi.nlm.nih.gov/genomes/all/GCF/000/412/775/GCF_000412775.1_ASM41277v1 |
| private | 45 | No |  |  | ST20120276 | Contig |  |  |  |
| private | 45 | No |  |  | ST20120283 | Contig |  |  |  |
| private | 45 | No |  |  | ST20120303 | Contig |  |  |  |
| private | 45 | No |  |  | ST20152505 | Contig |  |  |  |

Abbreviation: ST, Sequence Type; MRSA, Methicillin-Resistant *S. aureus*; Wgs, Whole genome sequencing; Seq rel date, Sequence release date; CG, Complete Genome. In bold, the genomes used as reference for each CC.

**Table S1. Characterization of the genomes used for the SNP based phylogenetic analysis**
